# Mapping the Evidence for Bioactive Compound-Based Adjunctive Therapies in Canine Ocular Diseases: A Scoping Review

**DOI:** 10.3390/vetsci13101013

**Published:** 2026-09-25

**Authors:** Yubin Bae, Yoonmi Jang, Seoyi Sim, Suji Choi

**Affiliations:** 1 Department of Animal Health and Welfare, College of Healthcare and Biotechnology, Semyung University, Jecheon 27136, Republic of Korea; bin_2@semyung.ac.kr (Y.B.); yuudll0u0@semyung.ac.kr (Y.J.); 2023196021@semyung.ac.kr (S.S.); 2 Interdisciplinary Major in AI-Assisted Natural Product Development, College of Korean Medicine, Semyung University, Jecheon 27136, Republic of Korea; 3 Division of Pharmacology, College of Korean Medicine, Semyung University, Jecheon 27136, Republic of Korea

**Keywords:** canine, ocular disease, bioactive compound, nutraceutical, keratoconjunctivitis sicca, cataract, glaucoma, antioxidant

## Abstract

Bioactive compounds are widely used as adjunctive therapies for canine ocular disease, yet the supporting evidence has not been systematically mapped. This scoping review characterised its distribution, design and direction. PubMed and CAB Abstracts were searched, supplemented by Google Scholar and backward citation searching; 1350 records were screened and the 50 included studies were charted by compound class, disease category and study design. They addressed keratoconjunctivitis sicca; the cornea and ocular surface; cataracts and lenses; retinal disease; and glaucoma, the last with only a single study. Thirteen were therapeutic trials in dogs with naturally occurring disease, seven of them in keratoconjunctivitis sicca; eleven further studies used healthy or purpose-bred dogs or induced models. The remaining evidence rested mainly on in vitro work, deficiency observations and uncontrolled reports. Null or unfavourable results were reported for topical hyaluronic acid in corneal wound healing and for dietary docosahexaenoic acid in progressive rod–cone degeneration. Reports on one marketed supplement differed in direction; commercial interests were declared in some but not others, and their influence could not be determined. This evidence map delineates where canine research has accumulated and where it has not, providing a basis for prioritising controlled trials with concurrent safety assessment.

## Simple Summary

Many dog owners give their pets supplements, such as antioxidants, vitamins, fish oil or plant extracts, in the hope of protecting their dog’s eyes, and veterinarians prescribe such products widely. It is not clear how much scientific evidence actually supports this practice, and which eye diseases have been studied and which have not. We searched the scientific literature systematically and examined 50 studies relevant to dogs. The clinical studies in dogs with naturally occurring disease are most numerous for dry eye; for cataracts, retinal disease and glaucoma, the evidence comes mainly from laboratory experiments and from dogs that were short of a nutrient rather than being treated with a supplement, and glaucoma is studied least of all. Several products did not work as expected, and harmful effects were reported for a few at higher doses. The reports on one commercial supplement disagreed with one another. This review maps where the evidence has accumulated, where it is limited and where it is absent, so that veterinarians and researchers can see what still needs to be tested. It does not assess how reliable the individual studies are.

## 1. Introduction

Chronic and vision-threatening ocular disease is a substantial clinical burden in companion dogs. Cataracts are among the most frequently encountered conditions and arise from inherited, diabetic, inflammatory and traumatic causes as well as from ageing; together, keratoconjunctivitis sicca, ulcerative and other ocular surface diseases, inherited and acquired retinal degeneration, and glaucoma account for much of the remaining caseload seen in small animal ophthalmology [1,2]. These conditions differ in their mechanisms and treatments, but they share a feature relevant to the present review: each is managed over long periods, and adjunctive products are widely used alongside conventional therapy.

The consequences extend beyond vision. Visual impairment in dogs is associated with increased anxiety and aggression and with reduced willingness to exercise, and may adversely affect the owner–animal relationship.

Phacoemulsification is the treatment of choice for canine cataracts, with reported success rates of 70–95% [2], and topical immunomodulation with ciclosporin A or tacrolimus is the standard of care for immune-mediated keratoconjunctivitis sicca (KCS) [3]. Nevertheless, therapeutic gaps persist in both conditions. In cataracts, a substantial proportion of patients present at a stage that does not yet warrant surgery, are geriatric with comorbidities that raise the anaesthetic risk, or belong to owners who decline surgical management [2]. In KCS, the response to immunosuppressive therapy varies considerably between individuals and treatment must be maintained indefinitely [3].

Against this background, bioactive compounds—antioxidants, vitamins, omega-3 fatty acids and plant extracts—are widely used as adjunctive therapies. Phytotherapeutic prescribing is already established in small animal practices in continental Europe [4]. Presumed neuroprotective agents are used in canine ocular disease across several clinical contexts, although the reported prevalence depends on the setting examined: in a survey addressing the prophylactic management of the normotensive fellow eye in dogs with primary glaucoma, 40% of respondents reported using a neuroprotectant agent [5], while a review written for practising clinicians describes grape seed extract, omega-3 fatty acids, lutein, zeaxanthin and vitamins as being used for degenerative retinal and optic nerve disease despite limited supporting evidence [6]. This clinical uptake is not matched by a correspondingly organised body of evidence, and the discrepancy between marketing claims and demonstrated efficacy has been explicitly criticised, most conspicuously in advertisements claiming complete resorption of mature cataracts by topical products [6].

Two narrative reviews have addressed related ground. One contrasted the benefits and the misinformation surrounding nutraceuticals in canine and feline ocular disease [6]; the other summarised the therapeutic options for canine KCS [3]. Neither reported a search strategy. The former devoted a substantial portion to feline herpesvirus, and the latter spanned immunosuppressants, blood products and stem cells rather than focusing on bioactive compounds. A 2024 review published in this journal surveyed plant extracts in dogs and cats across gastrointestinal, immune, cardiovascular and redox domains, but cited only two ophthalmic studies among its references [7]. The ocular application of bioactive compounds in dogs has therefore not been systematically charted.

The objective of this scoping review was to map the available literature and to identify where evidence has accumulated, where it is limited and where it is absent. The review asked which bioactive compounds have been applied to which canine ocular diseases, how the resulting literature is distributed across study designs and what the characteristics of those designs are, and what has been reported regarding safety as distinct from efficacy. Because no formal appraisal of methodological quality, risk of bias or certainty of evidence was undertaken, study design is described here as a characteristic of the literature rather than as a level of evidence.

## 2. Materials and Methods

### 2.1. Study Design

This review was conducted as a scoping review and reported in accordance with the PRISMA Extension for Scoping Reviews (PRISMA-ScR) [8]. Given the heterogeneity of interventions, outcome measures and study designs, a quantitative synthesis was not considered appropriate and no meta-analysis was performed. No protocol was registered in advance. PROSPERO does not accept scoping reviews for registration, and registration in a general-purpose registry, such as the Open Science Framework, was not undertaken; this is acknowledged among the limitations. A completed PRISMA-ScR checklist is provided as Supplementary Material (Table S1).

### 2.2. Operational Definition

For the purposes of this review, a bioactive compound was defined operationally as any of the following: extracts and fractions derived from plants, algae or fungi; nutrient-based supplements, including vitamins, minerals, amino acids and fatty acids; and biological materials of animal origin, such as amniotic and placental extracts, used for their bioactive constituents. Conventional pharmaceutical agents, surgical procedures and devices were outside this definition unless a bioactive constituent was the component under evaluation.

Purified single-molecule immunosuppressants of microbial origin that are licensed and marketed as prescription pharmaceuticals (ciclosporin A, tacrolimus, sirolimus, pimecrolimus), cell-based therapies and blood products were excluded, notwithstanding their natural derivation. The criterion applied was function rather than origin: licensed pharmacological agents with an established ophthalmic indication were excluded regardless of their natural derivation, because the question addressed here concerns adjunctive bioactive use rather than prescription pharmacotherapy. Studies combining such agents with a bioactive compound were included when the bioactive intervention was itself evaluated.

### 2.3. Information Sources and Search Strategy

The primary search was conducted in PubMed. CAB Abstracts was searched as a veterinary-specific supplementary source. Google Scholar was consulted as an additional source: one topic search sorted by relevance was screened for the first 100 records, and seven title-restricted (allintitle) searches were run and screened in full. The searches were performed on 23 July 2026. Scopus and Web of Science were not searched because neither is subscribed to by the authors’ institution.

The PubMed search was developed through three iterations of increasing sensitivity; the final strategy combined controlled vocabulary with free-text terms:

((“Dogs”[Mesh] OR dog[tiab] OR dogs[tiab] OR canine[tiab] OR canines[tiab])
AND (“Eye Diseases”[Mesh] OR “Eye”[Mesh] OR “Eye Diseases, Hereditary”[Mesh]
   OR eye[tiab] OR ocular[tiab] OR ophthalm*[tiab] OR cataract*[tiab]
   OR retina*[tiab] OR glaucoma[tiab] OR cornea*[tiab] OR conjunctiv*[tiab]
   OR uveitis[tiab] OR lens[tiab] OR tear*[tiab] OR lacrimal[tiab])
AND (“Antioxidants”[Mesh] OR “Dietary Supplements”[Mesh] OR “Plant Preparations”[Mesh]
   OR “Plants, Medicinal”[Mesh] OR “Complementary Therapies”[Mesh]
   OR “Phytotherapy”[Mesh] OR “Biological Products”[Mesh] OR “Vitamins”[Mesh]
   OR “Micronutrients”[Mesh] OR “Fatty Acids”[Mesh] OR “Diet”[Mesh]
   OR “Animal Feed”[Mesh] OR “Trace Elements”[Mesh]
   OR antioxidant*[tiab] OR supplement*[tiab] OR nutraceutical*[tiab]
   OR extract*[tiab] OR “natural product*”[tiab] OR phytochemical*[tiab]
   OR herbal[tiab] OR botanical[tiab] OR diet*[tiab] OR nutrition*[tiab]))

The CAB Abstracts strategy used CABI thesaurus descriptors:

(od:(dogs) OR ti:(dog OR dogs OR canine))
AND de:(“eye diseases” OR “eye” OR “eyes” OR “cataract” OR “glaucoma”
   OR “cornea” OR “retina” OR “conjunctivitis” OR “uveitis”
   OR “keratoconjunctivitis sicca” OR “keratitis” OR “lens” OR “tears”)
AND de:(“antioxidants” OR “plant extracts” OR “medicinal plants” OR “plant products”
   OR “dietary supplements” OR “feed supplements” OR “vitamins”
   OR “trace elements” OR “fatty acids” OR “polyunsaturated fatty acids”
   OR “nutrition” OR “diets” OR “herbal drugs” OR “phytotherapy”
   OR “bioactive compounds” OR “plant preparations”)

In addition to the searches above, the literature previously compiled by the investigators and one-step backward citation searching of included reviews and key primary studies were used as other sources. Chained citation searching beyond one step was not undertaken. These records were assembled by the investigators before the present review as part of their ongoing clinical and research interest in canine ocular disease, through non-systematic reading, citation following and conference attendance rather than through a documented search strategy. No search string or search date was recorded for these sources, and they are therefore not independently reproducible; this is addressed in the Limitations. The screening of this record set is described in Section 2.5.

### 2.4. Eligibility Criteria

Studies were eligible if they concerned a bioactive compound, as defined in Section 2.2, in dogs or in canine-derived cells, tissues, body fluids or microbial isolates, whether as an intervention material or as a test system, and reported an ocular outcome. Two kinds of records satisfied this requirement: studies in which a bioactive compound was administered or applied as an intervention, and observational studies in which the nutritional or endogenous status of such a compound was related to an ocular disease or an ocular outcome measure. The latter were retained because deficiency observations constitute a substantial part of the canine literature in this field and were one of the phenomena this map was designed to locate; they were charted as observational studies and were not counted as therapeutic evidence at any point. An ocular outcome was defined as any measurement made of the eye or of ocular tissue, including: vision; electroretinography; tear parameters; intraocular pressure; and corneal, retinal or lenticular findings. Studies reporting only systemic measurements, such as serum or plasma concentrations, without relating them to an ocular outcome, were not considered to report an ocular outcome. Eligible records were published in English in a peer-reviewed journal as an original article or as a review.

Records were excluded if they exclusively concerned a non-canine species or materials (retained as background where mechanistically relevant); if neither an intervention nor a relationship between compound status and an ocular outcome was reported, as in studies measuring an endogenous constituent solely as a physiological response to a surgical procedure; if they were conference abstracts, regulatory opinions or special-issue announcements; if they were not published in English; or if neither an abstract nor the full text could be obtained. Chapters of monographic book series that are indexed as serials by the National Library of Medicine, such as *Advances in Experimental Medicine and Biology*, were treated as journal articles and were eligible.

Narrative reviews were retained as included records because one objective of this evidence map was to document where the field has already been synthesised. They were charted as a separate study-design category and are identified as such in the evidence map and the distribution of study designs presented in Section 3, so thatthe volume of primary empirical evidence can be distinguished from secondary commentary. Reviews were counted as records in the numerical summaries but were not treated as independent sources of primary data at any point in the Results or Discussion.

Where an in vitro study used a canine-derived intervention material together with a non-canine test system, or a canine-derived test system with a non-canine intervention, the record was retained; this is addressed in the Limitations.

### 2.5. Selection Process

The records were deduplicated and screened based on title and abstract. Records in which ocular and bioactive terms co-occurred only incidentally were identified and excluded with documented reasons; the principal sources of such false positives were routine ophthalmic examination endpoints in beagle toxicology studies, incidental ocular symptoms in canine allergy research, and feed-additive regulatory opinions. Screening was performed by three reviewers. For the set of records compiled by the investigators before the database searches, each reviewer assessed all 177 records independently; agreement among the three reviewers was 86.4% (153/177), with a Fleiss κ of 0.774, and the corresponding author reviewed all judgements and made the final decisions. This screening was completed on 10 February 2026, and 20 of the studies included in the present review originated from this source. The records identified through the database searches were divided among the same three reviewers; each record was screened by one reviewer, with the corresponding author reviewing all screening decisions. Independent duplicate screening was therefore applied only to the pre-compiled record set, and overall inter-rater agreement across the full search yield could not be quantified.

During the present revision, every included record was re-assessed against the eligibility criteria. Each record was evaluated independently by two of the three reviewers using a standardised charting form covering species and material, administration of the intervention, ocular outcome, disease category, study design and route of administration; the corresponding author adjudicated discordant judgements. Full texts were available for 38 of the 50 records retained as included studies after re-screening; for the remaining 12 records, eligibility and classification were re-assessed using the available abstract and published bibliographic information, and the same constraint applied to a proportion of the records reclassified as background literature. Eleven records that had been counted as included studies were reclassified as background literature, two records were reassigned to a different disease category, and one record could not be assigned on the basis of the sources available and is reported as indeterminable in Figure 1. The availability of the full text for each record is recorded in Table S2.

### 2.6. Data Charting

The following were extracted: bibliographic details, class of bioactive compound, disease category, study design, subjects (species, number of animals or cell type), route of administration, and direction of principal findings. Study designs were categorised as therapeutic clinical trial, animal experiment, in vitro/ex vivo, observational study, case report, review or survey, and the same seven categories are used throughout the text, tables and figures, although only those categories represented among the included studies appear in the figures. A therapeutic clinical trial was defined as the administration of an intervention to dogs with naturally occurring ocular disease with clinical outcomes measured. Animal experiment covered experimentally induced models, studies in healthy or purpose-bred dogs, feeding trials and perioperative physiological measurements. In vitro/ex vivo covered work on cells or excised tissue. Observational study covered measurement or observation without an investigator-assigned intervention, including retrospective clinical evaluations. For reports containing more than one species or study design, classification was based on the component that generated the canine ocular findings. The complete charting table is provided as Supplementary Material (Table S2).

## 3. Results

### 3.1. Selection Results

Database searching yielded 1073 records from PubMed and 227 from CAB Abstracts; 62 of the CAB records were also retrieved by PubMed, leaving 165 unique CAB records and a database subtotal of 1238. Other sources contributed the 177 records previously compiled by the investigators, seven records identified through one-step backward citation searching and six records identified through Google Scholar. Of the previously compiled records, 102 were not retrieved by either database search. Of the records identified by the other methods, a further three were duplicated CAB Abstracts records whose titles had been truncated in the exported list and whose identity was only established at this stage, and these were not counted again, leaving five new records from citation searching and five from Google Scholar. A total of 1350 records were therefore screened based on their title and abstract, of which 1188 were excluded. Of the 162 reports sought for retrieval, four could not be obtained and 158 were assessed for eligibility. Fifty studies met the inclusion criteria (Figure 1). One hundred and eight reports were excluded as studies: 69 were retained as background or mechanistic literature and are cited where relevant, 34 did not meet the operational definition of a bioactive intervention or reported no ocular outcome, four could not be resolved from the available text, and one was a duplicate report of an included study. Eleven of the records retained as background literature [4,9,10,11,12,13,14,15,16,17,18], and one of the records reported as indeterminable [19], were reclassified from the included set during the re-screening described in Section 2.5.

### 3.2. Temporal and Disease Distribution

The included literature spanned from 1981 to 2026, with 21 of the 50 studies (42%) published since 2020 (Figure 2). Their distribution across disease categories was uneven: cornea and ocular surface disease and retinal disease each accounted for 13 studies, followed by cataracts and lenses (n = 10) and keratoconjunctivitis sicca (n = 9). Three studies were charted as ocular surface pharmacology and one as cross-cutting; glaucoma was represented by a single study. The characteristics of all 50 included studies are summarised in Table 1.

### 3.3. Evidence Map by Compound Class and Disease

Figure 3 presents the distribution of studies across compound classes and disease categories. Long-chain n-3 fatty acids (n = 4), vitamin E (n = 4) and taurine (n = 3) together account for 11 of the 13 retinal studies, largely through deficiency observations and dietary supplementation experiments. Fatty acids (n = 3) and hyaluronic acid (n = 2) account for five of the nine keratoconjunctivitis sicca studies. Plant extracts and polyphenols (n = 6) and animal-derived extracts (n = 4) together account for 10 of the 13 cornea and ocular surface studies, most of which have appeared since 2016. Cataract and lens studies are spread thinly across six compound classes, the largest being carnosine derivatives (n = 3) and multi-antioxidant blends (n = 2). Most cells in the matrix are empty, and no cell has more than six studies. The sparsest disease category is glaucoma, where a single plant-derived alkaloid appears [64]. Of the 50 included records, five are narrative reviews and 45 are primary empirical studies; the number of reviews contributing to each cell is shown in parentheses in Figure 3, so that the volume of primary empirical evidence can be distinguished from secondary commentary.

Three included studies do not map onto a disease category in the therapeutic sense. The aqueous leaf extract of Ottonia martiana was evaluated for corneal anaesthesia in healthy beagles rather than for the treatment of disease [66]; three sodium hyaluronate-based artificial tear formulations were evaluated for their immediate effect on tear film osmolality in healthy brachycephalic dogs [67]; and three traditional herbal extracts were evaluated for tear staining in dogs predisposed to it rather than for keratoconjunctivitis sicca [65]. All three satisfy the operational definition of a bioactive compound and report a canine ocular outcome, and are therefore retained, but they are charted as a separate category (ocular surface pharmacology) so that they do not inflate the apparent volume of disease-specific therapeutic evidence.

### 3.4. Distribution of Study Designs

Figure 4 shows the study designs by disease category. Thirteen of the 50 studies were therapeutic clinical trials, that is, studies in which an intervention was administered to dogs with naturally occurring ocular disease and the clinical outcomes were measured. These were concentrated in keratoconjunctivitis sicca, which accounted for seven of the 13 [20,21,22,23,24,25,27]. A further eleven in vivo studies were conducted in healthy or purpose-bred dogs or in experimentally induced models; these addressed physiological or preventive questions rather than the treatment of established disease, and are counted separately here for that reason.

In the other disease categories, the study designs addressed different questions. Regarding cornea and ocular surface disease, nine of the 13 studies were in vitro [28,29,30,32,33,34,35,38,40]. Regarding retinal disease, 10 of the 13 studies were observational studies or dietary experiments, including experimentally induced deficiency models, rather than therapeutic trials [51,53,54,55,56,57,58,59,60,61], and only one study administered an intervention to dogs with naturally occurring retinal disease [62]. The cataract and lens studies contained five therapeutic clinical trials, comprising one placebo-controlled double-masked pilot [43], one randomised topical study [47], one preliminary study of oral alpha-lipoic acid [42] and two perioperative studies [41,46]; the remaining cataract evidence rests on in vitro work in canine lens epithelial cells [44,48] and on a retrospective series [45]. Glaucoma was represented by a single acute experiment in normal dogs [64], with no therapeutic clinical trial at all.

### 3.5. Null and Unfavourable Findings

Several studies reported null or unfavourable results. Topical 0.2% hyaluronic acid did not accelerate corneal epithelialisation and did not alter MMP-2/9 expression in a masked randomised study in beagles [39], and fisetin added to ciclosporin did not restore tear production, altering only tear film MMP-9 [27]. In the lens, grape seed extract reduced reactive oxygen species in canine lens epithelial cells but did not prevent ex vivo posterior capsule opacification; lutein behaved pro-oxidatively; fish oil increased posterior capsule opacification [48]; and oral antioxidant supplementation showed no delaying effect on incipient cataracts, with benefit confined to immature cataracts [45]. In the retina, diets enriched in docosahexaenoic acid failed to correct the progressive rod–cone degeneration phenotype [62].

### 3.6. Safety Findings

No included study reported systematic adverse event ascertainment, so the following are reported safety signals rather than the products of safety evaluations.

Among the included studies, aloe vera did not affect the viability of canine corneal cells but slowed corneal fibroblast wound closure at higher concentrations and increased the collagenase-mediated degradation of type IV collagen, a basement membrane constituent [40]. For a pequi peel extract active against methicillin-resistant Staphylococcus pseudintermedius isolated from canine ocular surfaces, the antimicrobially effective concentration (2.2%) [34] exceeded, by approximately one order of magnitude, the non-irritating concentration subsequently determined for the same extract in a non-canine irritation assay (0.24%); the latter was not an included study and is discussed in Section 4.4. In contrast, an aqueous leaf extract of Ottonia martiana reduced corneal sensitivity in healthy beagles without the reduction in tear production or the conjunctival hyperaemia observed with the synthetic comparator proxymetacaine [66].

Two further canine toxicity reports were identified during the review but did not meet the eligibility criteria because they reported no ocular outcome; they are retained as background literature and are considered in Section 4.4 rather than here.

### 3.7. Contradictory Evidence for a Single Commercial Product

One oral multi-antioxidant formulation marketed for canine ocular health has been evaluated in reports that differ in the direction of their findings (Table 2). The included reports focused on different populations, at different stages of disease and had different outcome measures, so the differences between them are not necessarily contradictory; one report in Table 2 is a rodent study that did not meet the eligibility criteria of this review and is identified as such. The interpretation of these reports is considered in Section 4.5.

## 4. Discussion

### 4.1. Principal Finding

The central finding of this evidence map is asymmetry. The available clinical studies in canine ophthalmology are concentrated on one condition. Keratoconjunctivitis sicca accounts for seven of the 13 therapeutic clinical trials identified, and for that condition, fatty acids and hyaluronate-based preparations have been examined repeatedly with concordant results [20,22,23,24,25]. No other disease category contains more than five such trials; retinal disease contains one and glaucoma contains none. This review counted studies and did not appraise their quality, so the concentration on keratoconjunctivitis sicca should be read as accumulated clinical attention rather than as demonstrated efficacy. For cataracts, retinal degeneration and glaucoma, the literature rests largely on in vitro experiments, deficiency observations and uncontrolled or perioperative series, notwithstanding widespread clinical use [5,6].

### 4.2. Route of Administration as a Hypothesis

One hypothesis that emerges from the evidence map, rather than an explanation established by it, concerns route of administration. The lens is avascular and derives its nutrients exclusively from the aqueous humour, and orally administered antioxidants may therefore fail to reach therapeutic concentrations across the blood–aqueous barrier. Oxidative injury in canine senile cataracts has been reported to be local, with elevated malondialdehyde and depleted reduced glutathione in the aqueous humour and lens [69], while systemic markers of lipid peroxidation are also altered in affected dogs [70]. In cataractous poodles, the activities of several erythrocyte antioxidant enzymes were significantly lower than in unaffected dogs, whereas plasma and aqueous-humour ascorbic acid concentrations did not differ between groups [71].

The observation that recent positive findings cluster around topically applied preparations for the ocular surface [20,24,31,36], whereas orally administered products for the lens and retina have yielded equivocal results [45,62], is compatible with this hypothesis. It is equally compatible, however, with differences in disease, compounds, study design, outcome measures and the methodological characteristics of the underlying studies, none of which were appraised here. The same contrast is evident within the single commercial formulation discussed in Section 3.7, where a topical preparation outperformed the oral product in rodent models [68]; topical aldose reductase inhibition has likewise been shown to modify cataract development in dogs [72]. Route of administration is therefore proposed as a hypothesis generated by this evidence map and as a variable that future comparative work should address explicitly, rather than as a demonstrated determinant of efficacy. The pharmacokinetic demonstration of ocular tissue penetration should, in either case, be regarded as a prerequisite rather than an optional addition; such a demonstration was reported for a traditional herbal formulation, whose constituents were detected in canine ocular tissue after three days of oral dosing, although no ocular outcome was measured [13].

### 4.3. Species Extrapolation

Findings in rodents cannot be assumed to transfer to dogs. Expression of the lenticular antioxidant system differs between dogs, rats, marmosets and rabbits, and this difference was proposed as a determinant of species predilection to oxidative lens injury [73]. The divergent results obtained for the same commercial supplement in dogs and in rats [43,68] illustrate the practical consequence. The point is not confined to the lens: a work on taurine and tapetal structure, which examined normal and taurine-depleted cats together with a naturally occurring tapetal abnormality in the beagle dog [61], and works on retinal fatty acid handling in inherited degeneration [59,62] indicate that both the target tissue and its nutrient economy differ from the rodent models in which most mechanistic screening is performed.

### 4.4. Safety Is Not Established by Natural Origin

The assumption that compounds of natural origin are inherently safe is not supported. The findings summarised in Section 3.6 indicate that the constraint on translation is frequently not the discovery of activity but the demonstration of a therapeutic window: for the pequi peel extract, the antimicrobially effective and the non-irritating concentrations differ by approximately an order of magnitude [10,34]. Aloe vera, widely regarded as benign, increased type IV collagen degradation in canine corneal cells [40].

Three further sources bear on safety but did not meet the eligibility criteria of this review and are cited here as background literature. Total lignans from Arctium fruit, a source previously reported to inhibit streptozotocin-induced diabetic retinopathy, caused death in half of the beagles exposed to 28 days of repeated oral dosing at the highest tested dose, with lesions in the digestive tract, heart, liver and kidneys [74]. Green tea polyphenol exposure in fasted dogs is associated with gastrointestinal irritation, weight loss and mortality [75]. Neither report measured an ocular outcome. The HET-CAM irritation assay that established the non-irritating concentration of the pequi peel extract used embryonated chicken eggs rather than canine material and was likewise not an included study [10]. The absence of a systematic safety literature is itself a finding of this review: no included study was designed primarily to establish an ocular safety margin.

### 4.5. Independence of the Evidence Base

The pattern described in Section 3.7 warrants explicit comment. The reports on this product differ in the direction of their findings. Commercial affiliations or patent interests were declared in some of these reports and not in others; one report declared no competing interest [45]. Whether this influenced the results cannot be determined from the available reports. This is a post hoc observation rather than a pre-planned analysis, and it is reported here as a reason for independent replication rather than as a judgement on any individual study. A fourth report on the same formulation, a conference abstract describing eight dogs against historical controls, met the exclusion criteria of this review and was not charted; it is noted here because it compared censored observation times with event times by an unpaired t-test. This concern is not merely hypothetical for practitioners: presumed neuroprotective agents are used by a substantial proportion of practitioners in at least one clinical context [5], and are described as being used for degenerative retinal and optic nerve disease despite limited evidence [6]. Comparable scrutiny has been applied to the human ophthalmic nutraceutical literature, where reviews have had to set out the concentrations actually used in preclinical and clinical studies alongside the limitations of the supporting evidence [76]. Whether such concentrations are attained in ocular tissue after systemic administration has itself been raised as a reason for developing topical rather than oral formulations [68].

A related feature of this literature is the concentration of key findings within a small number of research groups. Reports evaluating carnosine derivatives for canine cataracts have originated predominantly from one team [47,49,50], and in vitro studies of canine lens epithelial responses largely from a single laboratory [44,48]; canine amniotic membrane preparations for the ocular surface are likewise the output of one centre [35,38]. Oral multi-antioxidant formulations have been investigated by different research groups [43,45], although differences in populations, disease stages and outcome measures preclude an inference of independent replication. Hyaluronate-based preparations for keratoconjunctivitis sicca [20,24] and dietary long-chain n-3 fatty acids for retinal function [53,54,59] have similarly been examined at more than one institution. It should be noted throughout that investigation by more than one research centre is not the same as independent replication of a given effect, since the studies concerned differed in their preparations, doses and outcome measures; Table 3 therefore records whether a compound class has been investigated by more than one centre, and not whether any specific finding has been replicated.

### 4.6. Absence of Evidence in Glaucoma

Glaucoma is the clearest gap in the map. Only one included study addressed it: an experimental demonstration of dose-dependent intraocular pressure reduction by a plant-derived muscarinic alkaloid in normal dogs [64]. A pilot quantification of taurine in the plasma and aqueous humour was conducted in dogs without ocular disease and is cited here as background literature providing ocular biomarker rather than therapeutic evidence [18]. This is difficult to reconcile with the mechanistic literature, in which oxidative stress is increased in the retinas of dogs with acute glaucoma [77] and is repeatedly proposed as a contributor to retinal ganglion cell loss [78]. The disparity between the reported use of neuroprotective supplementation in canine glaucoma [5] and the near absence of canine efficacy data makes glaucoma one of the most prominent research gaps identified by this review, alongside the substantial gaps also apparent for cataracts and retinal disease.

### 4.7. Candidate Compounds by Disease Category

Because this review did not appraise methodological quality, risk of bias or certainty of evidence, the compounds identified below are those for which canine evidence has accumulated in the greatest volume and with the most consistent direction, and for which findings have been reported by more than one research group, where applicable. They are not ranked by demonstrated efficacy, and the accompanying limitations are as important as the findings themselves (Table 3).

For keratoconjunctivitis sicca, hyaluronate-based preparations and oral omega-3 fatty acids have been examined in several therapeutic clinical trials with concordant direction, and work on hyaluronate has been reported by more than one research centre. For cornea and ocular surface disease, canine amniotic membrane preparations and plant-derived antioxidants have generated the largest volume of in vitro evidence, but the amniotic membrane work has originated predominantly from a single group and, in two studies, used a human rather than a canine test system.

For cataracts and lenses, carnosine derivatives and oral multi-antioxidant formulations have been evaluated most often, but the carnosine literature has originated largely from one research team, the multi-antioxidant reports differ in the direction of their findings and differ in their declared interests, and the in vitro lens epithelial work is predominantly the output of a single laboratory. For retinal disease, long-chain n-3 fatty acids, vitamin E and taurine account for most of the evidence, but this consists chiefly of deficiency observations and dietary supplementation experiments in healthy or purpose-bred animals rather than treatment of established disease, and the one controlled dietary intervention in affected dogs was negative.

For glaucoma, no bioactive compound currently evaluated in dogs can be considered a promising candidate: the single included study was an acute intraocular pressure experiment in normal dogs, and no controlled clinical evidence exists. The same conclusion applies to cataracts, where no compound has been shown to alter the course of established disease in a controlled canine trial that has been independently replicated.

### 4.8. Limitations

First, the search was restricted to English-language publications; domestic and other non-English literature was not included. Second, CAB Abstracts could not be exported in bulk owing to institutional subscription limitations, and its records were screened at title level with individual full-text retrieval attempted for relevant items. Overlap between the CAB and PubMed record sets could not be quantified electronically because the CAB records carry no PubMed identifier, and duplicate removal between these two sources therefore relied on manual title matching. Third, only two bibliographic databases were searched; Scopus and Web of Science were not available at the authors’ institution, and Google Scholar was used as a supplementary source screened only to a predefined depth rather than exhaustively. Fourth, four records could not be assessed because neither an abstract nor the full text could be obtained. Fifth, veterinary journals published in some regions are incompletely indexed in the databases searched, and one-step citation searching may not have identified all such literature; the Latin American ocular phytotherapy studies retrieved in this way [10,34,66] suggest that further such work exists. Sixth, a substantial proportion of included studies were in vitro experiments or small uncontrolled clinical studies, and heterogeneity of interventions, doses and outcome measures precluded quantitative synthesis. Seventh, independent duplicate screening was applied only to the set of records compiled by the investigators before the database searches; the records identified through the database searches were screened by a single reviewer with adjudication by the corresponding author, so the overall inter-rater agreement across the full search yield could not be quantified. Eighth, the records compiled by the investigators before the review contributed a substantial proportion of the screening pool but were assembled without a documented search strategy; this source is not independently reproducible and may preferentially capture the literature already familiar to the review team. Ninth, the review protocol was not prospectively registered. Because the operational definition of a bioactive compound, the assignment of disease categories and the classification of the direction of findings all involve judgements that are to some degree subjective, the absence of a registered protocol limits the demonstrable reproducibility of these decisions; the criteria applied and the re-screening carried out during revision are reported in full in Section 2.4 and Section 2.5 and in Table S2. Tenth, two included in vitro studies evaluated canine-derived intervention material in a human corneal epithelial cell line rather than in canine cells [35,38]; they were retained because the intervention material was canine, but the test system was not, and this should be borne in mind when interpreting the ocular surface evidence. Consistent with scoping review methodology, a formal risk-of-bias appraisal was not undertaken [8]; this review mapped the distribution of published evidence rather than evaluating methodological quality or certainty of evidence, and the counts reported throughout should be read accordingly. Eleventh, observational studies relating nutritional or endogenous compound status to an ocular outcome were retained as included records; they document where deficiency-based evidence exists but do not test a therapeutic intervention, and the counts reported here should be read with that distinction in mind. Twelfth, full texts could not be obtained for all records. Of the 50 studies included after re-screening, 38 were re-assessed against the full text and 12 against the available abstract and published bibliographic information; the same constraint applied to a proportion of the records reclassified as background literature. For records without full text, the study-level information retained from the original charting could not in all cases be independently re-verified against the full report, and one record could not be assigned to either group and is reported as indeterminable. The availability of the full text for each record is recorded in Table S2.

## 5. Conclusions

Bioactive compounds are used extensively as adjunctive therapy in canine ophthalmology, but the supporting evidence is concentrated in a single disease category and thins rapidly elsewhere. Controlled canine trials are required for cataracts, retinal degeneration and glaucoma in particular; safety assessments should be conducted concurrently rather than presumed; pharmacokinetic evidence of ocular penetration should accompany efficacy claims, with particular attention to route of administration; and the independence of study sponsorship warrants closer attention. For practitioners, the immediate implication is that adjunctive bioactive use in canine keratoconjunctivitis sicca is supported by a larger number of clinical studies in dogs with naturally occurring disease than any other category, whereas comparable evidence for cataracts, retinal degeneration and glaucoma is limited or absent, and discussions with owners should reflect that difference. For researchers, the priority is to convert the mechanistic work that already exists in these three domains into controlled canine trials. This evidence map is intended to make these gaps explicit and to inform the prioritisation of future translational research in veterinary ophthalmology.

## Figures and Tables

**Figure 1:**
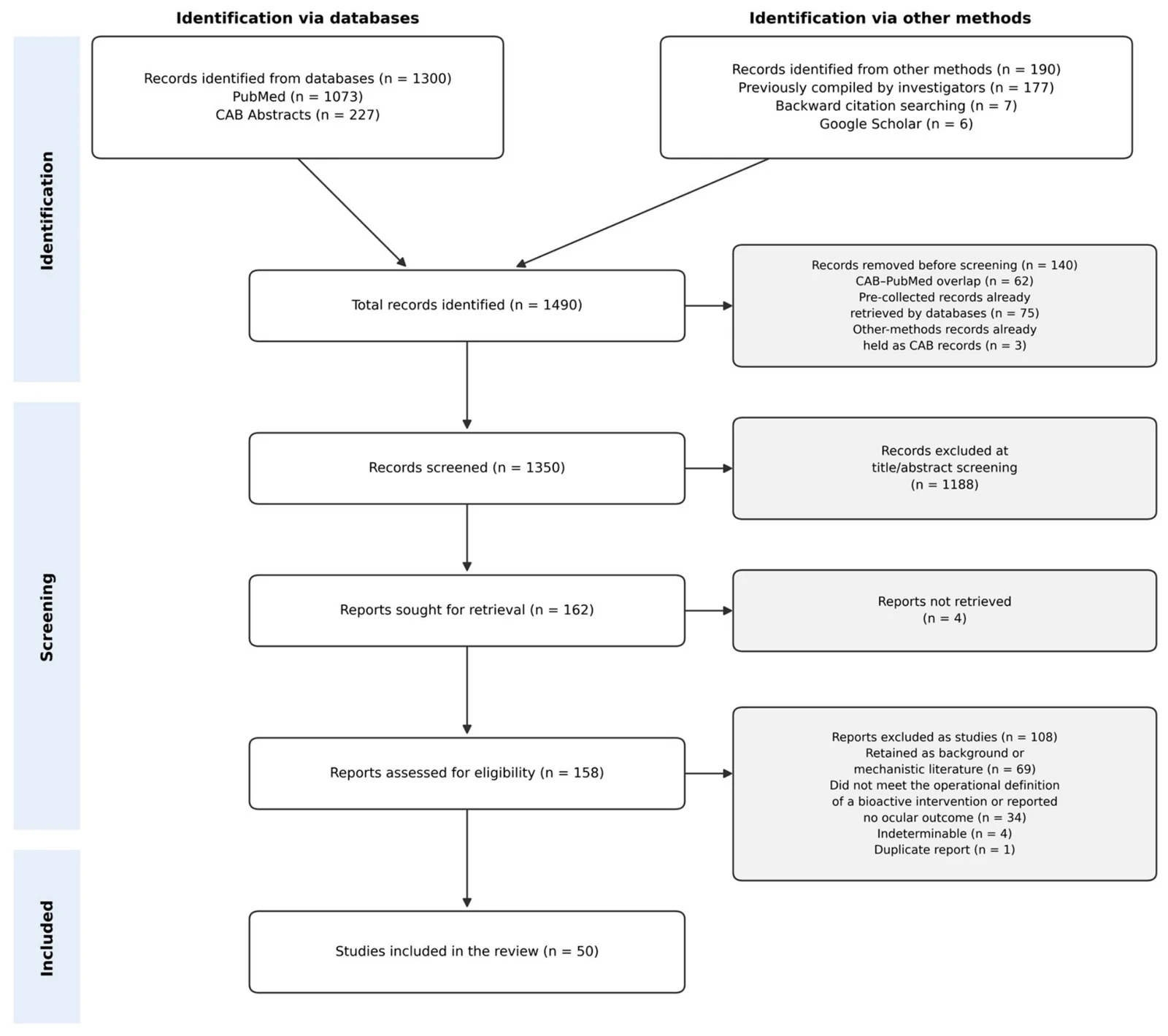
PRISMA-ScR flow of study identification, screening and inclusion.

**Figure 2:**
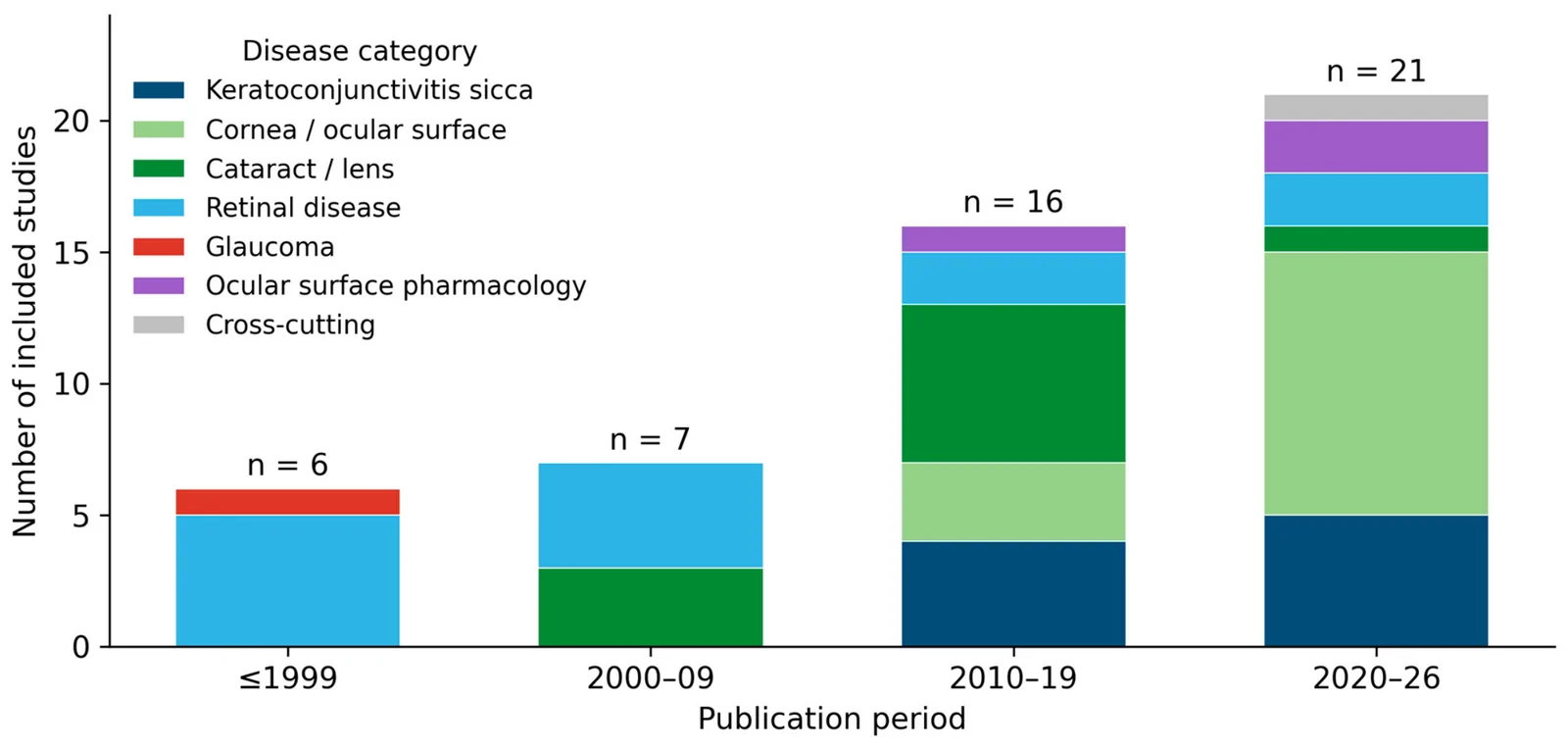
Distribution of the 50 included studies by publication period, stacked by disease category. Totals are shown above each bar.

**Figure 3:**
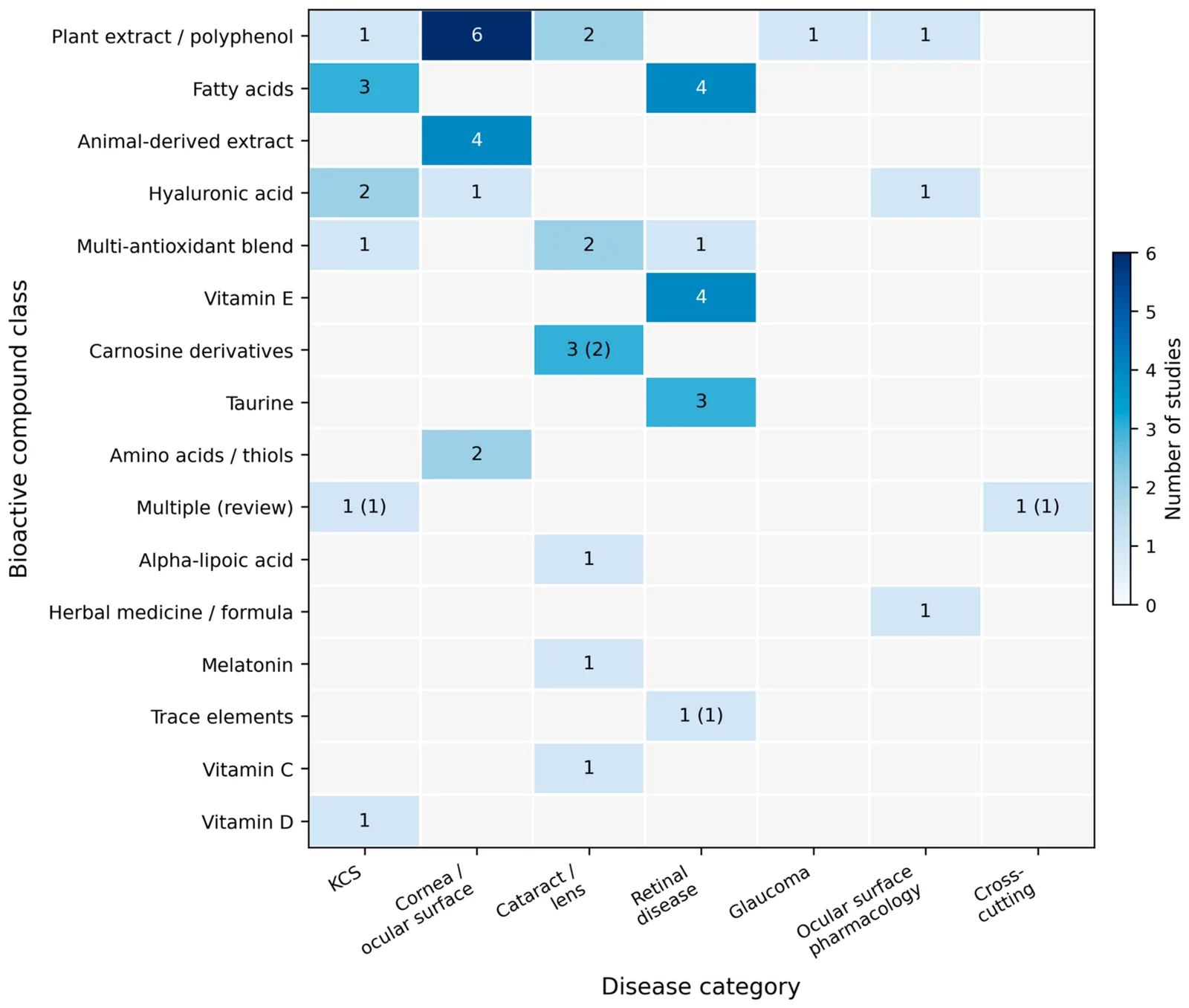
Evidence map of the 50 included studies by compound class and disease category. Counts are shown within each cell; where a cell includes narrative reviews, the number of reviews is given in parentheses. Empty cells denote combinations for which no canine study was identified. Of the 50 included records, 5 are narrative reviews and 45 are primary empirical studies. KCS, keratoconjunctivitis sicca.

**Figure 4:**
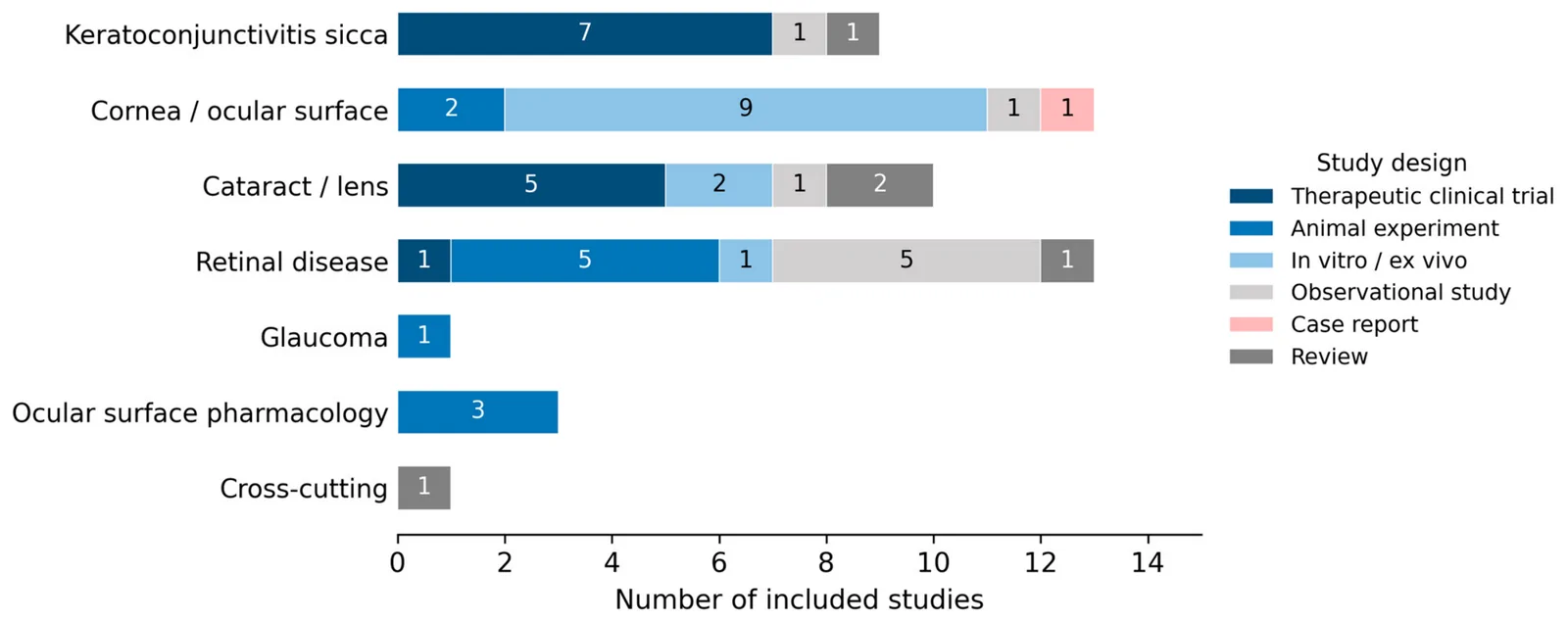
Distribution of study designs within each disease category. Counts are shown within each segment. Study design categories are defined in Section 2.6.

**Table 1:** Characteristics of the 50 included studies, grouped by disease category and ordered by the direction of the reported findings. Direction labels describe the direction of the findings as classified by the authors and do not represent an assessment of methodological quality, risk of bias or certainty of evidence. Full study-level charting, including whether the full text of each record was available at re-screening, is provided in Table S2.

Compound Class	Study Design and Subjects	Route	Principal Finding	Direction	Ref.
**Keratoconjunctivitis sicca (n = 9)**					
Cross-linked sodium hyaluronate	Pilot clinical trial, 19 dogs, 30 days	Topical ocular	Ocular clinical score, TBUT, STT-1 and quality of life improved	Positive	[20]
Periocular fatty acid cream with 0.15% HA	Exploratory clinical study, 10 dogs with idiopathic KCS	Periocular	Applied after failure of tobramycin; tear parameters improved	Positive	[21]
Oral omega-3 (varying EPA:DHA) with antioxidants	Comparative clinical trial, 45 dogs, 6 months	Oral	Clinical indices improved over topical tacrolimus alone	Positive	[22]
Nutraceutical diet (anti-inflammatory, antioxidant)	Randomised clinical trial, 50 dogs with immune-mediated KCS, 60 days	Dietary	Tear production, conjunctival inflammation, keratinisation, pigmentation and mucoid discharge all improved	Positive	[23]
Cross-linked HA hydrogel (xCMHA-S)	Masked controlled study, dogs with KCS	Topical ocular	Superior to standard HA for hyperaemia, irritation and discharge	Positive	[24]
Oral omega-3, with homologous PRP	Randomised clinical trial, 22 dogs (44 eyes), 6 months	Periocular	STT-1 rose in both arms; combination superior for TBUT and goblet cell density from 3 months	Partly positive	[25]
Vitamin D (serum 25-hydroxyvitamin D)	Observational study, 61 dogs (122 eyes)	—	25(OH)D higher in dogs with normal STT-1; no association with TBUT	Descriptive	[26]
Fisetin 0.1% topical, added to ciclosporin	Clinical trial, dogs with dry eye disease	Topical ocular	Tear production not restored at 7 or 14 days; tear film MMP-9 reduced at 14 days	Null	[27]
Multiple (narrative review)	Review of canine KCS therapeutics	—	Tear substitutes, secretagogues, fatty acids, blood products and stem cells compared	Review	[3]
**Cornea and ocular surface (n = 13)**					
Berberine (alkaloid)	In vitro, canine corneal epithelial cells	In vitro exposure	NOD2–RIPK2/NF-κB activation and NLRP3 pyroptosis inhibited; Nrf2 response restored	Positive	[28]
Dandelion extract with lysozyme	In vitro, canine corneal fibroblasts	In vitro exposure	IL-6, IL-8 and TNF-α reduced; SOD, catalase and glutathione increased	Positive	[29]
Eugenol with biogenic silver nanoparticles	In vitro, *S. pseudintermedius* from canine KCS	In vitro exposure	Synergistic antibacterial effect	Positive	[30]
Equine placenta extract	Case report, 2 dogs	Topical ocular	Corneal lesions unresponsive to HA and antibiotics improved	Positive	[31]
N-acetylcysteine, dimethyl fumarate	In vitro, canine corneal epithelial cells	In vitro exposure	NLRP3–HMGB1-mediated pyroptosis and oxidative injury attenuated	Positive	[32]
Glutamine	In vitro, canine corneal epithelium, LPS model	In vitro exposure	Inflammation and oxidative stress alleviated	Positive	[33]
Pequi peel extract	In vitro antibacterial testing, isolates from 21 dogs	In vitro exposure	2.2% inhibited MRSP growth; pulp hexane extract ineffective	Positive	[34]
Canine amniotic membrane extracts	In vitro	In vitro exposure	HGF, TIMP and related mediators identified; wound healing efficacy confirmed	Positive	[35]
Onion extract	Animal experiment, 24 canine eyes after lamellar keratectomy	Topical ocular	Corneal haze reduced from day 14; comparable to prednisolone	Positive	[36]
Amniotic membrane extract eye drops	Retrospective clinical study, dogs with SCCED	Topical ocular	Healing time and re-treatment rate compared with debridement alone	Partly positive	[37]
Canine amniotic membrane and extracts	In vitro, 14 canine amniotic membranes	In vitro exposure	Proteomic profiles and wound healing properties compared across preparations	Descriptive	[38]
Topical HA 0.2%	Masked randomised trial, 6 beagles, experimental epithelial defect	Topical ocular	No difference from control in clinical score, epithelialisation or MMP-2/9	Null	[39]
*Aloe vera* (0–2 mg/mL)	In vitro, canine corneal epithelial cells and fibroblasts	In vitro exposure	Viability unaffected; fibroblast wound closure delayed above 175 µg/mL; type IV collagen degradation increased	Unfavourable	[40]
**Cataracts and lenses (n = 10)**					
Intracameral ascorbic acid	Clinical trial, 20 dogs (40 eyes) undergoing phacoemulsification	Intracameral	Corneal endothelial cell loss and morphometric deterioration attenuated	Positive	[41]
Oral alpha-lipoic acid	Preliminary clinical study, diabetic dogs	Oral	Fewer and later lens opacities than with ascorbate plus tocopherol	Positive	[42]
Oral multi-antioxidant formulation	Placebo-controlled double-masked pilot, 15 vs. 15 diabetic dogs	Oral	Median time to lens change 261 vs. 84 days; reversal reported in 2 dogs	Positive	[43]
Grape polyphenols (proanthocyanidin, resveratrol)	In vitro, canine LEC	In vitro exposure	Reactive oxygen species reduced at 30 min; MAPK and PI3K signalling altered	Positive	[44]
Oral multi-antioxidant formulations	Retrospective observational study, 112 and 60 dogs	Oral	No effect on incipient cataract; progression delayed in immature cataract	Partly positive	[45]
Melatonin	Clinical trial, 11 diabetic and 13 healthy dogs	Oral	Post-phacoemulsification inflammatory complications assessed	Partly positive	[46]
Topical N-acetylcarnosine 2% with antioxidants	Randomised clinical trial, 30 dogs, 8 weeks	Topical ocular	Opacity change tracked by retroillumination photography	Partly positive	[47]
Grape seed extract, lutein, fish oil	In vitro/ex vivo, canine LEC	In vitro exposure	Grape seed reduced reactive oxygen species; lutein pro-oxidative; fish oil increased PCO; all reduced viability	Unfavourable	[48]
N-acetylcarnosine (drug delivery)	Review	—	Lens epithelial telomere and telomerase targeting discussed	Review	[49]
N-acetylcarnosine	Review, human and canine	—	Lipid peroxidation and N-acetylcarnosine in age-related cataract	Review	[50]
**Retinal disease (n = 13)**					
Multi-antioxidant supplement	Animal experiment, 12 healthy beagles, 6 months	Oral	ERG a-wave amplitude and cone responses increased; refractive error change reduced	Positive	[51]
Alpha-tocopherol	In vitro, canine retinal homogenate	In vitro exposure	Iron/ascorbate-induced lipid peroxidation fully inhibited at 1 µmol/mg	Positive	[52]
Long-chain n-3 versus alpha-linolenic acid	Randomised feeding trial, 12 dams and their puppies	Dietary	Puppy ERG a-wave amplitude higher and implicit time shorter with marine oil than with alpha-linolenic acid	Positive	[53]
DHA-fortified diet	Animal experiment (feeding trial), 48 beagle puppies, 8–52 weeks	Dietary	Visual discrimination learning, memory and ERG function assessed by DHA level	Partly positive	[54]
Vitamin E (TTPA genotype)	Genetic and observational study, English cocker spaniels	—	TTPA deletion associated with vitamin E deficiency retinopathy	Descriptive	[55]
Taurine	Observational cohort study, English cocker spaniels	—	Five dogs with low blood taurine concentration had retinal abnormalities; blood taurine concentration was also associated with dietary methionine, protein source and age	Descriptive	[56]
Taurine	Observational study, Newfoundlands	—	Among nine taurine-deficient dogs examined ophthalmically, two had fundus abnormalities, including one with generalised retinal degeneration; a direct causal relationship with taurine deficiency could not be established	Descriptive	[57]
Vitamin E	Observational study, hunting dogs	—	Sensory retinal degeneration with tissue vitamin E at 10–40% of controls	Descriptive	[58]
Maternal dietary n-3	Feeding experiment, puppies at 50 days	Dietary	Retinal 22:6n-3 reduced and 20:4n-6, 22:5n-6 raised on low n-3 diets	Descriptive	[59]
Vitamin E	Animal experiment; dogs on purified diets varying in vitamin E, selenium and methionine	Dietary	Deficiency retinopathy characterised	Descriptive	[60]
Taurine	Basic study of cats and beagles; canine component observational	—	Relationship between taurine and tapetal structure	Descriptive	[61]
DHA-enriched diet	Feeding trial, dogs with progressive rod–cone degeneration	Dietary	Phenotype not corrected	Null	[62]
Zinc	Review	—	Zinc concentrated in retina and choroid; deficiency alters ocular structure and electrophysiology	Review	[63]
**Glaucoma (n = 1)**					
Muscarinic alkaloid from *Trophis racemosa*	Animal experiment, 5 normal dogs	Topical ocular	Dose-dependent IOP reduction of 6.6–15.7 mmHg; blocked by atropine and pirenzepine	Positive	[64]
**Ocular surface pharmacology (non-disease outcome) (n = 3)**					
Herbal extracts (*Chrysanthemum morifolium*, Cassia semen, Poria cocos), 0.5% dietary	Animal experiment (feeding trial), 40 poodles and bichons	Dietary	Tear-staining index with concurrent iron status, immune and antioxidant markers	Positive	[65]
*Ottonia martiana* aqueous leaf extract	Animal experiment, 45 healthy beagles in 3 groups	Topical ocular	Corneal sensitivity reduced as with proxymetacaine, but without the fall in tear production or the conjunctival hyperaemia seen with proxymetacaine	Positive (tolerability)	[66]
HA (0.4–1.6%), vitamin B12 artificial tears	Animal experiment, 40 healthy brachycephalic dogs	Topical ocular	Immediate change in tear film osmolality after instillation	Descriptive	[67]
**Cross-cutting (n = 1)**					
Multiple (narrative review)	Review of nutraceuticals in veterinary ophthalmology	—	Benefits distinguished from marketing claims across cataract, retinal degeneration and ocular surface disease	Review	[6]

Abbreviations: KCS, keratoconjunctivitis sicca; HA, hyaluronic acid; STT, Schirmer tear test; TBUT, tear film break-up time; ERG, electroretinography; LEC, lens epithelial cell; PRP, platelet-rich plasma; MRSP, methicillin-resistant *Staphylococcus pseudintermedius*; SCCED, spontaneous chronic corneal epithelial defect; IOP, intraocular pressure; PCO, posterior capsule opacification.

**Table 2:** Reports evaluating a single commercially marketed oral multi-antioxidant formulation for canine ocular disease.

Design	Subjects	Direction	Considerations	Ref.
Placebo-controlled, double-masked pilot	15 vs. 15 diabetic dogs	Positive	Non-indexed journal; co-author affiliated with the distributor	[43]
Retrospective observational study	112 and 60 dogs	Partly positive	No effect on incipient cataracts	[45]
Comparative study in streptozotocin-diabetic and ultraviolet-exposed rats; the marketed formulation was included as one oral comparator arm	Rodent	Negative	Not an included study (rodent); cited as background. Author holds the patent for a competing topical formulation	[68]

**Table 3:** Compounds with the greatest accumulated canine evidence in each disease category, with the predominant evidence type, whether the compound class has been investigated by more than one research centre, and the principal limitations. Counts in the evidence-type column refer to the entire disease category and not exclusively to the compounds listed. Investigation by more than one research centre is not equivalent to independent replication of a given effect. The compounds listed are not ranked by their demonstrated efficacy; no appraisal of methodological quality, risk of bias or certainty of evidence was undertaken, and pharmacokinetic and ocular-exposure data were not systematically collected in this review.

Disease Category	Compounds with the Greatest Accumulated Canine Evidence	Predominant Evidence Type in the Category	Investigated by More than One Research Centre	Principal Limitations
Keratoconjunctivitis sicca	Hyaluronate-based preparations; oral omega-3 fatty acids	Therapeutic clinical trials (n = 7)	Yes	Small samples; short follow-up; heterogeneous outcome measures
Cornea and ocular surface	Canine amniotic membrane preparations; plant-derived antioxidants	In vitro (n = 9)	Partly	Amniotic membrane work predominantly from one group; two studies used a human corneal cell line
Cataracts and lenses	Carnosine derivatives; oral multi-antioxidant formulations	Therapeutic clinical trials (n = 5); in vitro (n = 2)	Varies by compound class: yes for oral multi-antioxidant formulations [43,45], no for carnosine derivatives	Carnosine literature from a single team; multi-antioxidant reports differ in direction and differ in declared interests; lens epithelial work from a single laboratory
Retinal disease	Long-chain n-3 fatty acids; vitamin E; taurine	Observational and dietary experiments (n = 10)	Yes	Almost entirely deficiency observations or feeding studies in healthy animals; the one dietary intervention in affected dogs was negative
Glaucoma	None	Single acute experiment in normal dogs (n = 1)	No	No controlled clinical evidence; no compound can be considered a promising candidate

## Data Availability

All data charted in this review are available in the Supplementary Materials. No new data were created.

## Supplementary Materials

The following supporting information can be downloaded at: https://www.mdpi.com/article/10.3390/vetsci13101013/s1. Table S1: PRISMA-ScR checklist; Table S2: Data charting table, comprising the included studies, the records retained as background literature, other manuscript citations, and explanatory notes.

## Abbreviations

The following abbreviations are used in this manuscript:

DHA	docosahexaenoic acid
EPA	eicosapentaenoic acid
ERG	electroretinography
HA	hyaluronic acid
HET-CAM	hen’s egg test on the chorioallantoic membrane
IOP	intraocular pressure
KCS	keratoconjunctivitis sicca
LEC	lens epithelial cells
MMP	matrix metalloproteinase
MRSP	methicillin-resistant *Staphylococcus pseudintermedius*
PCO	posterior capsule opacification
PRISMA-ScR	Preferred Reporting Items for Systematic Reviews and Meta-Analyses Extension for Scoping Reviews
PRP	platelet-rich plasma
SCCED	spontaneous chronic corneal epithelial defect
STT	Schirmer tear test
TBUT	tear film break-up time
TTPA	alpha-tocopherol transfer protein
