# Supplementary material for: Mapping the Evidence for Bioactive Compound-Based Adjunctive Therapies in Canine Ocular Diseases: A Scoping Review
Source: Vet Sci. 2026 Sep 25;13(10):1013. doi: 10.3390/vetsci13101013 (PMC13709839; doi:10.3390/vetsci13101013)
Supplement: Supplementary file 1 [file vetsci-13-01013-s001.zip › Table_S1_PRISMA_ScR_checklist.pdf]

Table S1. PRISMA-ScR Checklist

Supplementary material to: *Mapping the Evidence for Bioactive Compound-Based Adjunctive Therapies in Canine Ocular Diseases: A Scoping Review.*

Checklist items follow Tricco et al., Ann. Intern. Med. 2018, 169, 467–473. "Reported in" refers to the section of the revised manuscript in which the item is addressed. Section numbering follows the revised version, in which the selection results were moved from Section 2.7 to Section 3.1 and a new Section 4.7 was added, so that the Limitations are now Section 4.8.

#	Section / item	Checklist item	Reported in
**TITLE**			
1	Title	Identify the report as a scoping review.	Title
**ABSTRACT**			
2	Structured summary	Provide a structured summary including, as applicable, background, objectives, eligibility criteria, sources of evidence, charting methods, results and conclusions.	Abstract
**INTRODUCTION**			
3	Rationale	Describe the rationale for the review in the context of what is already known; explain why the review questions lend themselves to a scoping review approach.	Section 1
4	Objectives	Provide an explicit statement of the questions and objectives, with reference to their key elements and other relevant key elements used to conceptualise the review questions and/or objectives.	Section 1 (questions 1–3)
**METHODS**			
5	Protocol and registration	Indicate whether a review protocol exists, state if and where it can be accessed, and if available provide registration information including the	Section 2.1 — no protocol was registered; this is stated explicitly and repeated in Section 4.8

#	Section / item	Checklist item	Reported in
		registration number.	
6	Eligibility criteria	Specify characteristics of the sources of evidence used as eligibility criteria and provide a rationale.	Sections 2.2 and 2.4
7	Information sources	Describe all information sources in the search and the date the most recent search was executed.	Section 2.3
8	Search	Present the full electronic search strategy for at least 1 database, including any limits used, such that it could be repeated.	Section 2.3 (full PubMed and CAB Abstracts strategies)
9	Selection of sources of evidence	State the process for selecting sources of evidence (i.e., screening and eligibility) included in the scoping review.	Section 2.5 (including the re-screening carried out during revision)
10	Data charting process	Describe the methods of charting data from the included sources of evidence and any processes for obtaining and confirming data from investigators.	Section 2.6
11	Data items	List and define all variables for which data were sought and any assumptions and simplifications made.	Section 2.6; Table S2 (S2_Notes sheet)
12	Critical appraisal of individual sources of evidence	If done, provide a rationale for conducting a critical appraisal; describe the methods used and how this information was used in any data synthesis.	Not performed; rationale in Section 4.8
13	Synthesis of results	Describe the methods of handling and summarising the data that were charted.	Section 2.1 (no meta-analysis); Sections 3.1–3.7
**RESULTS**			
14	Selection of sources of evidence	Give numbers of sources of evidence screened, assessed for eligibility, and included in the review, with reasons for exclusions at each stage, ideally	Section 3.1; Figure 1

#	Section / item	Checklist item	Reported in
		using a flow diagram.	
15	Characteristics of sources of evidence	For each source of evidence, present characteristics for which data were charted and provide the citations.	Table 1; Table S2
16	Critical appraisal within sources of evidence	If done, present data on critical appraisal of included sources of evidence.	Not applicable
17	Results of individual sources of evidence	For each included source of evidence, present the relevant data that were charted and that relate to the review questions and objectives.	Table 1; Sections 3.5–3.7
18	Synthesis of results	Summarise and/or present the charting results as they relate to the review questions and objectives.	Sections 3.2–3.4; Figures 2–4
**DISCUSSION**			
19	Summary of evidence	Summarise the main results (including an overview of concepts, themes, and types of evidence available), link to the review questions and objectives, and consider the relevance to key groups.	Sections 4.1–4.7
20	Limitations	Discuss the limitations of the scoping review process.	Section 4.8
21	Conclusions	Provide a general interpretation of the results with respect to the review questions and objectives, as well as potential implications and/or next steps.	Section 5
**FUNDING**			
22	Funding	Describe sources of funding for the included sources of evidence, as well as sources of funding for the scoping review. Describe the role of the funders of the scoping review.	Funding statement; commercial interests of included sources are addressed in Sections 3.7 and 4.5
